# Giant Cell Arteritis Presenting As Pyrexia of Unknown Origin: Diagnosis Made by Bilateral Periluminal Dark Halo Sign on Color Doppler Ultrasound

**DOI:** 10.7759/cureus.86134

**Published:** 2025-06-16

**Authors:** Rehan Siddique, Sally Alezergawi, Aminah Patel, Amber Khan

**Affiliations:** 1 Department of Diabetes and Endocrinology, Salford Royal NHS Foundation Trust, Manchester, GBR

**Keywords:** giant cell arteritis, halo sign, pyrexia of unknown origin, temporal artery ultrasound, vasculitis

## Abstract

Giant cell arteritis (GCA), a vasculitis of medium- and large-sized arteries, frequently manifests with symptoms such as headaches, soreness in the scalp, and vision abnormalities. Pyrexia is an uncommon symptom and can cause a delay in diagnosis. We describe a 76-year-old woman of Chinese ethnicity who did not exhibit the typical clinical signs of GCA but instead presented with a generalized lethargy, nausea, dizziness, and a persistent fever. Numerous tests, including autoimmune, neoplastic, and viral workups, came up negative. Pyrexia did not settle despite using broad-spectrum antibiotics. A temporal artery Doppler ultrasound was performed to assess the condition further, as inflammatory markers (erythrocyte sedimentation rate, ESR, and C-reactive protein) remained high. The ultrasound Doppler results showed bilateral halo signs that are very specific for GCA. Upon starting corticosteroid treatment, there was a rapid improvement in fever and ESR. This case underscores the importance of considering GCA as a differential diagnosis for pyrexia of unknown origin, particularly in the elderly. It highlights the utility of temporal artery ultrasound in facilitating timely diagnosis in atypical cases.

## Introduction

Giant cell arteritis (GCA) is a granulomatous vasculitis predominantly affecting medium- and large-sized arteries, typically seen in individuals over 50 years of age. Most often seen in people of Northern European heritage, it often manifests with symptoms including jaw claudication, scalp discomfort, temporal headache, and vision loss. However, GCA can sometimes appear atypically, making diagnosis extremely difficult. One uncommon manifestation of GCA is pyrexia of undetermined origin (PUO), which frequently results in a delayed diagnosis [1]. This case report emphasizes the significance of considering GCA as part of the differential diagnosis for persistent fever, even in the absence of traditional symptoms or findings. Moreover, the case highlights the importance of color Doppler ultrasonography in facilitating early diagnosis of GCA with high sensitivity and prompt treatment in cases where suspicion of GCA is low and the presentation is atypical [2].

## Case presentation

A 76-year-old woman presented to the emergency department with a one-week history of generalized fatigue, mild nonproductive cough, dizziness, and nausea. Of note, she was of Chinese ethnicity, which is considered atypical for GCA, a condition more commonly observed in Caucasian populations. Her only medication was atorvastatin for dyslipidemia. She reported no use of over-the-counter or herbal medications. There was no history of recent foreign travel.

On examination, she was febrile with a temperature of 37.9°C, which later on peaked to 38.4°C. General physical, cardiovascular, respiratory, and abdominal examinations were unremarkable.

Initial laboratory investigations revealed significantly elevated inflammatory markers and liver function tests (Table 1). Based on her presentation, she was treated empirically for infection of unknown origin with piperacillin-tazobactam, in line with the hospital’s local antibiotic policy.

**Table 1:** Initial tests including immunology screen CTD: connective tissue disease; MPO: myeloperoxidase; AI: antibody index; PR3: anti-proteinase 3; LK: liver-kidney; TTG IgA: tissue transglutaminase immunoglobulin A; ALT: alanine transaminase; ALP: alkaline phosphatase; INR: international normalized ratio

Blood tests	Result	Reference range
Hemoglobulin	99	130-180 g/L
White cell count	13.3	4.0-11.0 × 10^9^/L
Lymphocyte	1.2	1.0-4.0 × 10^9^/L
Neutrophil	13.3	1.8-7.5 × 10^9^/L
Eosinophils	0.1	0.0-0.4 × 10^9^/L
Platelet count	262	150-450 × 10^9^/L
C-reactive protein	299	<10.0 mg/L
Erythrocyte sedimentation rate	>120	1-7 mm/hour
Serum sodium	133	133-146 mmol/L
Serum potassium	4.3	3.5-5.3 mmol/L
Corrected calcium	2.28	2.20-2.60 mmol/L
Urea	4.5	2.5-7.8 mmol/L
Creatinine	40	65-104 umol/L
Estimated glomerular filtration rate	>90	>90 mL/minute/1.73 m^2^
Thyroid-stimulating hormone	0.62	10.0-20.0 pmol/L
Free T4	16.1	0.35-5.50 mU/L
Prolactin	127	38-420 mU/L
Serum cortisol	567	145-619 nmol/L
Luteinizing hormone	11.7	16.0-54.0 U/L
Follicle-stimulating hormone	23.1	23.0-116.0 U/L
C3 complement level	1.97	0.75-1.65 g/L
C4 complement level	0.32	0.14-0.54 g/L
Rheumatoid factor	<10	0.0-13.0 ku/L
BioPlex CTD screen	Negative	-
MPO BioPlex	<0.2	0.0-0.9 AI
Anti-centromere antibody	<0.2	0.0-0.9 AI
PR3 BioPlex	<0.2	0.0-0.9 AI
Smooth muscle antibody	Negative	-
LK microsomal antibody	Negative	-
Mitochondrial antibody	Negative	-
Gastric parietal antibody	Negative	-
Anti-TTG IgA antibodies	Negative	-
Epstein-Barr virus serology	Past infection	-
Liver screen and liver function tests	Hepatitis B core antibody positive. Bilirubin 18 (0-21 umol/L), ALT 55 (7-48 U/L), ALP 180 (40-129 U/L), albumin 35, and INR 1.0	-
Peripheral blood culture	No growth	-
Sputum sample (culture and sensitivity)	Regional flora isolated	-
Urine culture	Microscopy screen negative	-

Despite 48 hours of antibiotics, the patient remained febrile. A series of investigations, including a complete blood count, renal and liver function tests, blood and urine cultures, respiratory viral swabs, hepatitis virology screening, HIV testing, and a chest X-ray, were unremarkable. Ultrasound of the abdomen showed mild gallbladder inflammation. However, further evaluation with a focused gallbladder ultrasound and magnetic resonance cholangiopancreatography excluded cholecystitis as the source of her fever.

Given the unclear etiology, the cardiology team was consulted to rule out infective endocarditis, despite a low pretest probability based on Duke’s criteria. Both transthoracic and transoesophageal echocardiograms showed no evidence of vegetations.

Further history-taking revealed a mild headache and dizziness before admission, which the patient attributed to minor head trauma sustained when she struck her head on a bedside cabinet. A CT head was unremarkable, and subsequent MRI brain imaging identified a benign pituitary cyst with no signs of acute pathology. CT thorax, abdomen, and pelvis showed no source of infection. A whole-body fluorodeoxyglucose (FDG) positron-emission tomography scan was organized, which showed no FDG-avid focus of infection or inflammation.

The patient’s fever persisted for 20 days despite escalation of antibiotics to meropenem. At this point, the microbiology team recommended discontinuing antibiotics and monitoring her condition. At this point, C-reactive protein (CRP) was 117, and erythrocyte sedimentation rate (ESR) was >120. Three sets of peripheral blood cultures and urine cultures were negative. Rheumatology was consulted to evaluate for possible vasculitis, although there were no clinical features suggestive of the condition. Immunology tests, including antineutrophil cytoplasmic antibody (ANCA) and antinuclear antibody (ANA), were negative (Table 1).

**Figure 1:**
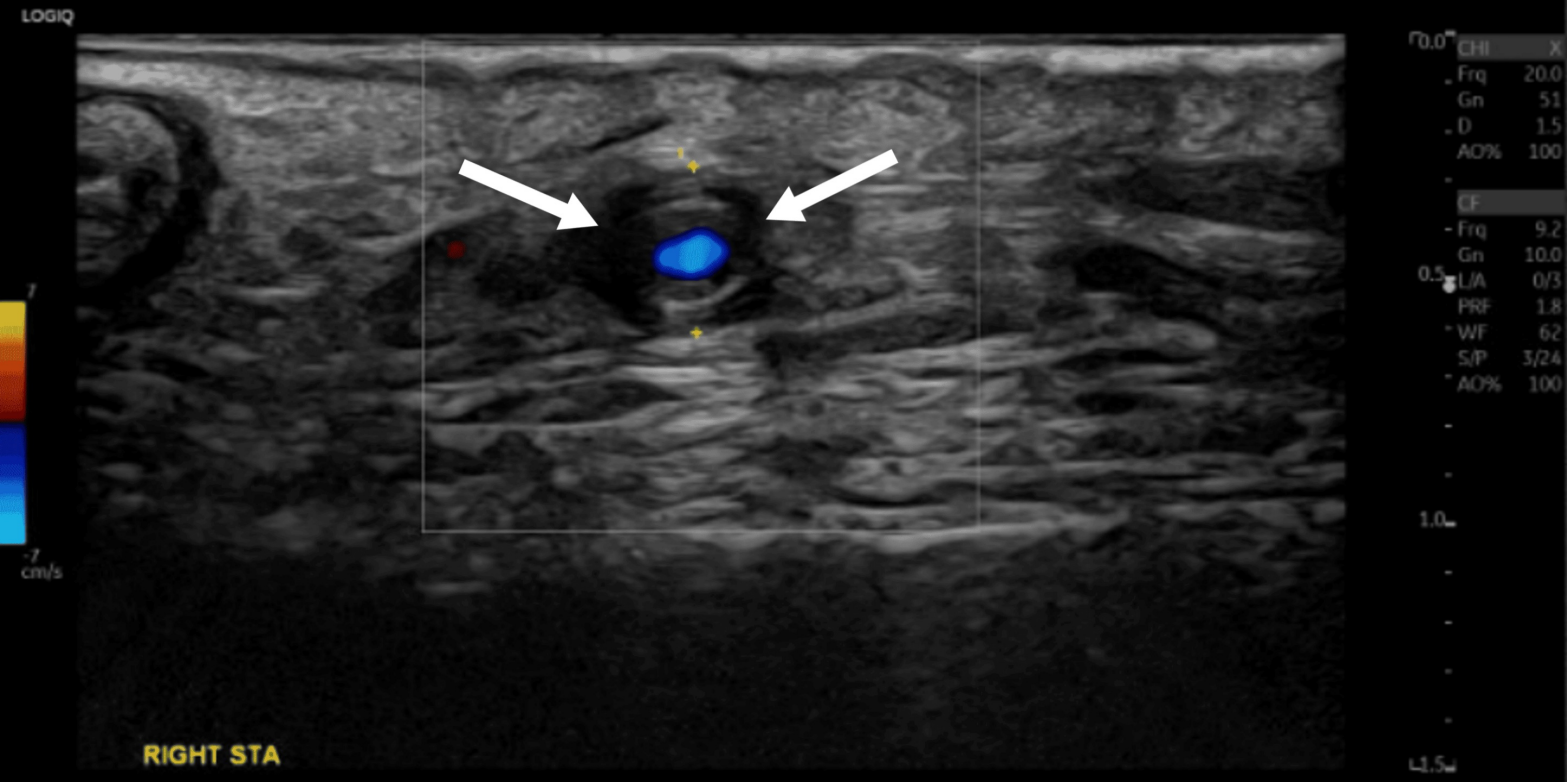
Temporal artery color Doppler ultrasound (right STA) Ultrasound images show a dark hypoechoic halo around the right STA lumen (arrows), representing the vessel wall inflammation STA: superficial temporal artery

**Figure 2:**
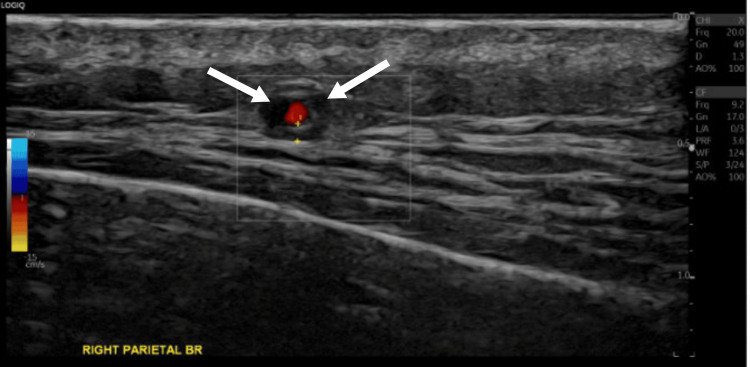
Temporal artery color Doppler ultrasound (right parietal branch) Ultrasound image showing marked hypoechoic "halo sign" (arrows) and circumferential wall thickening

**Figure 3:**
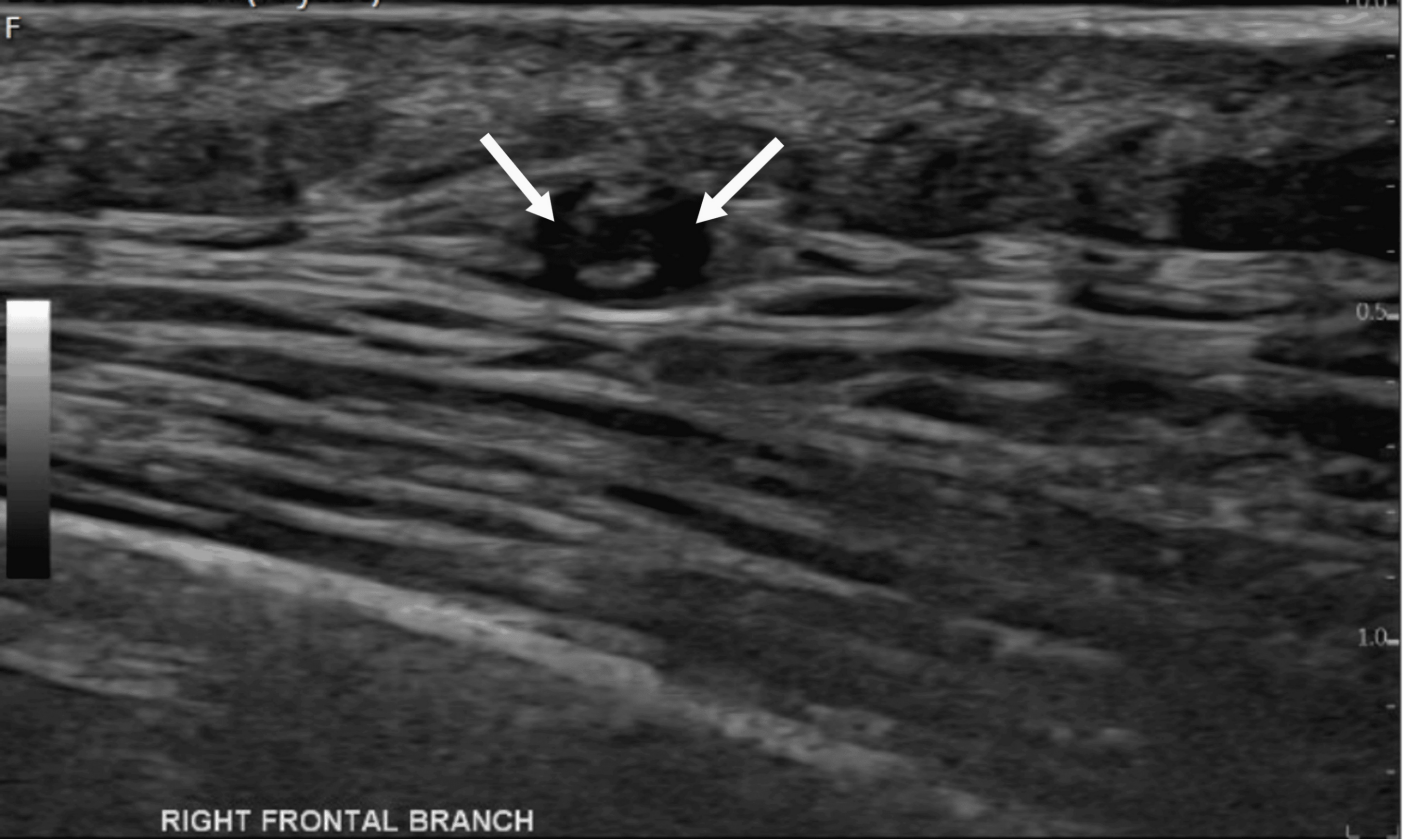
The right frontal branch US Doppler Positive compression sign, the hypoechoic area persists (arrow) during the compression maneuver of the vessel lumen US: ultrasound

**Figure 4:**
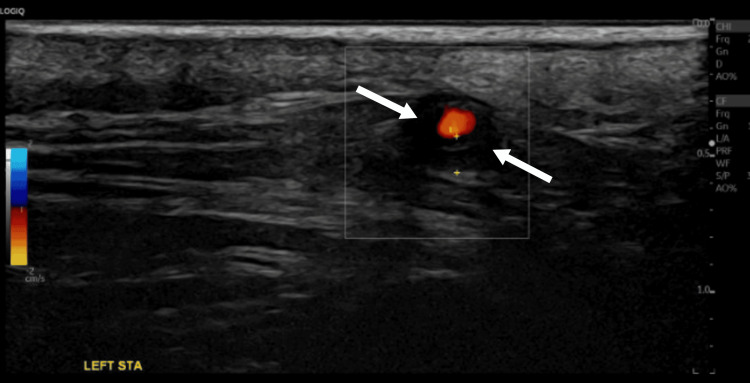
The left STA US Doppler Left STA showing circumferential hypoechoic wall thickening and periluminal dark "halo sign" (arrows) STA: superficial temporal artery; US: ultrasound

**Figure 5:**
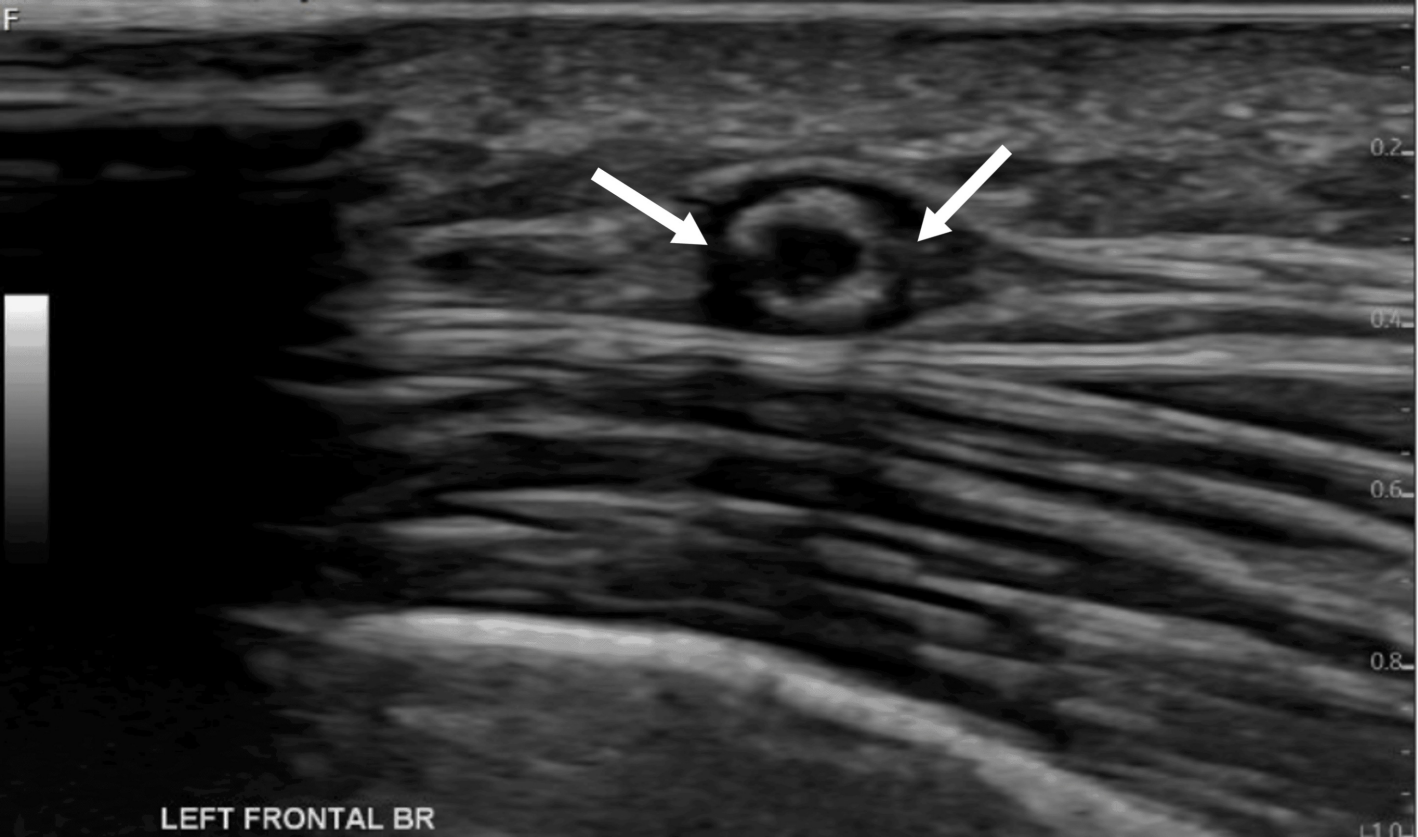
The left parietal branch US Doppler There is sonographic evidence of a dark periluminal halo sign (arrows), highly suggestive of giant cell arteritis US: ultrasound

**Figure 6:**
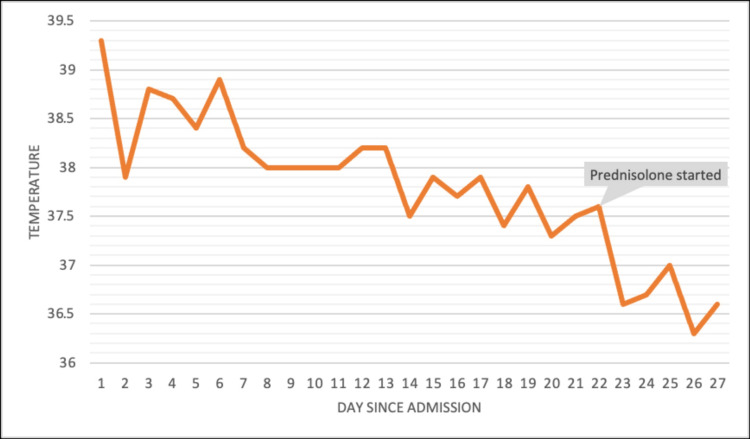
Trend of temperature throughout admission

The rheumatology team suggested a temporal artery ultrasound due to persistently high ESR. This revealed bilateral halo signs (Figures 1-5), a finding highly specific for GCA. Unfortunately, halo scores were not calculated, which has a prognostic value and would have helped to establish improvement on steroids. The patient was initiated on prednisolone 40 mg daily, resulting in marked symptomatic improvement within 48 hours. Pyrexia improved following initiation of steroids with no further temperature spikes (Figure 6). Subsequently, the patient was discharged home on a tapering dose of steroid, and follow-up was arranged under rheumatology.

## Discussion

The nonspecific initial presentation of fever, nausea, dizziness, and generalized exhaustion made it more difficult to diagnose an underlying inflammatory condition. Although PUO in older people may be linked to autoimmune, neoplastic, or viral diseases, the National Institute for Health and Care Excellence estimates that the incidence of GCA in the United Kingdom is around 2.2 per 10,000 person-years. GCA is more common in Northern European communities, such as the UK, than in Southern European ones, and it is more common in older adults, especially those over 50. GCA is nevertheless important to consider in patients presenting with PUO, even if the patient does not exhibit typical symptoms such as jaw claudication, vision loss, or soreness in the temporal arteries [1].

The fact that fever remained persistent even after receiving broad-spectrum antibiotics raises questions about noninfectious causes of PUO. Since rheumatology's involvement led to the crucial diagnostic discovery, this patient's case serves as further evidence of the need for interdisciplinary collaboration. The immunology tests (ANA and ANCA) that came back negative further diverted the workup from other systemic vasculitides, which usually involve small vessels. GCA might be indicated subtly by moderate, nonspecific symptoms, including headache and dizziness, which were initially mistaken for minor head injuries from a fall.

Temporal artery ultrasound has become an increasingly employed, noninvasive diagnostic method, especially with the identification of the "halo sign," which has a high specificity for GCA. The diagnostic approach to GCA has evolved in recent decades, with an increasing reliance on vascular imaging modalities, such as color duplex ultrasound (CDUS), especially in centers with experienced ultrasonographers. While temporal artery biopsy remains an important confirmatory tool, imaging is now often used as a first-line diagnostic step in clinical practice [1]. Historically, temporal artery biopsy has been the gold standard for diagnosis. However, the bilateral halo sign in this instance confirmed the diagnosis, which prompted the initiation of glucocorticoid medication and a swift improvement in clinical outcomes.

One of the most specific ultrasound findings for GCA is the "halo sign," which is a hypoechoic thickening of the arterial wall. The halo sign, especially when bilateral, is a powerful predictor of GCA. Temporal artery ultrasound has enough diagnostic accuracy to support its inclusion in clinical practice and future classification criteria, according to Aranda-Valera et al. [3]. Moreover, bilateral halo signals lessen the need for temporal artery biopsy, according to Santhanam and Mani [1], who recommended biopsy only when CDUS results are unclear. In a meta-analysis of 17 trials with 998 patients, Ball et al. further supported the diagnostic utility of duplex ultrasonography by showing that it had an 83% specificity and a 75% sensitivity for the halo sign compared to temporal artery biopsy [4]. The authors concluded that duplex imaging ought to be the initial investigation, with biopsy being saved for patients whose ultrasound results were negative or unclear. This easily accessible, noninvasive method is especially helpful for older individuals and those who are more likely to experience difficulties from invasive procedures.

In recent decades, the diagnostic approach to GCA has changed, with a greater emphasis on vascular imaging modalities such as CDUS, particularly in facilities with skilled ultrasonographers. In clinical practice, imaging is now frequently utilized as a first-line diagnostic step, and temporal artery biopsy is still a crucial confirmation tool [4]. Clinical and histological features, including age at onset, new-onset headache, high erythrocyte sedimentation rate (ESR), and temporal artery biopsy, were the main emphasis of the American College of Rheumatology's (ACR) initial diagnostic criteria set forth in 1990. A more thorough foundation for diagnosing GCA is offered by the 2022 ACR/European League Against Rheumatism (EULAR) revised classification criteria (Table 2), albeit given the increasing use of imaging modalities such as CDUS [5].

**Table 2:** 2022 American College of Rheumatology and EULAR classification criteria for giant cell arteritis CRP: C-reactive protein; US: ultrasound; FDG-PET: fluorodeoxyglucose positron-emission tomography; EULAR: European League Against Rheumatism

Absolute requirement	
Age ≥50 years at the time of diagnosis	
Additional clinical criteria	
Morning stiffness in the neck/shoulders	+2
Sudden visual loss	+3
Jaw or tongue claudication	+2
New temporal headache	+2
Scalp tenderness	+2
Abnormal examination of the temporal artery	+2
Laboratory, imaging, and biopsy criteria	
Maximum erythrocyte sedimentation rate ≥50 mm/hour or CRP ≥10 mg/L	+3
Positive temporal artery biopsy or halo sign on temporal artery US	+5
Bilateral axillary involvement	+2
FDG-PET activity throughout the aorta	+2

According to the revised ACR 2022 criteria, the updated criteria assign scores to 10 clinical, laboratory, imaging, and histopathological findings. Interestingly, a positive temporal artery biopsy or the observation of the halo sign on CDUS are credited with the highest score, indicating the excellent specificity of these results. Additional clinical criteria include morning stiffness in the neck or shoulders, jaw or tongue pain, and abrupt loss of vision. The scoring is further influenced by imaging findings of large-vessel involvement and laboratory thresholds for inflammatory markers such as ESR and CRP. A GCA diagnosis can be confirmed with a total score of six or higher [5].

In this case, the identification of bilateral halo signs on temporal artery ultrasound provided a definitive diagnosis, demonstrating the value of imaging-based criteria in clinical practice. The integration of imaging into the revised criteria aligns with the findings of studies such as those by Aranda-Valera et al. [3], Molina-Collada et al. [6], and Santhanam and Mani [1], which highlight the diagnostic accuracy of the halo sign and its potential to reduce the need for invasive biopsies. The revised criteria also emphasize the importance of a multidisciplinary approach, integrating clinical, laboratory, and imaging findings to optimize diagnostic accuracy and patient outcomes.

This case underscores the importance of adopting the revised ACR/EULAR criteria in clinical practice, particularly for atypical presentations such as PUO, and highlights the growing role of noninvasive imaging techniques as first-line diagnostic tools in GCA.

## Conclusions

This case contributes to the growing awareness of atypical GCA presentations, particularly in non-Caucasian populations and those without classical symptoms. Temporal artery ultrasound with color flow Doppler shall be considered while investigating PUO. Early diagnoses can improve patient outcomes and avoid potential consequences linked to delayed treatment, like irreversible vision loss, cerebrovascular problems, or aortic aneurysms. The cornerstone of care continues to be early identification and treatment with corticosteroids. A careful corticosteroid dose tapering plan is necessary to minimize long-term side effects. Active ongoing monitoring by rheumatology, in an outpatient setting, is required.
